# Sema4D Aggravated LPS-Induced Injury via Activation of the MAPK Signaling Pathway in ATDC5 Chondrocytes

**DOI:** 10.1155/2020/8691534

**Published:** 2020-04-25

**Authors:** Jinlai Lei, Yahui Fu, Yan Zhuang, Kun Zhang

**Affiliations:** Department of Orthopaedic Trauma, Honghui Hospital, Xi'an Jiaotong University, Xi'an, Shaanxi Province 710054, China

## Abstract

Osteoarthritis (OA) is the most common chronic degenerative joint disease, and it remains the main cause of chronic disability in elderly individuals. Sema4D (semaphorin 4D) is involved in the immune system and related to bone injury, osteoporosis, osteoblast differentiation, and rheumatoid arthritis. However, the role of Sema4D in OA remains unclear. Hence, the LPS-stimulated chondrocyte cell injury model was constructed in this study to investigate the role of Sema4D in OA development. The results showed that Sema4D was increased in LPS-treated ATDC5 cells, and the knockdown of Sema4D suppressed the decline of cell viability, the increase of cell apoptosis, and the increase of IL-6, IL-1*β*, and TNF-*α* secretion in ATDC5 cells induced by LPS. Meanwhile, Sema4D overexpression aggravated the cell injury triggered by LPS, and inhibiting Plexin B1 partly abolished the effect of Sema4D overexpression on LPS-induced chondrocyte injury. Furthermore, silencing of Sema4D blocked the activation of the MAPK pathway in LPS-stimulated ATDC5 cells. Enhanced Sema4D promoted the activation of the MAPK pathway in LPS-stimulated ATDC5 cells. What is more, inhibiting the MAPK signaling pathway abolished the promoting effect of Sema4D overexpression on LPS-induced chondrocyte injury. Therefore, our study suggested that the knockdown of Sema4D protects ATDC5 cells against LPS-induced injury through inactivation of the MAPK signaling pathway via interacting with Plexin B1.

## 1. Introduction

Osteoarthritis (OA) is the most common chronic degenerative joint disease, and it remains the main cause of chronic disability in elderly individuals [1]. This disease is characterized by sclerosis of the subchondral bone sclerosis, degeneration of articular cartilage, and synovium inflammation [2]. It is estimated that up to 240 million people suffer from OA around the world [1]. However, the available agents for OA therapy can only temporarily relieve symptoms and have many side-effects, due to incomplete understanding of the pathogenesis of OA. Hence, it is greatly needed to further explore the pathogenesis of OA and look for new targets for the prevention and treatment of OA.

Sema4D/CD100 (semaphorin 4D) belongs to the class 4 semaphorin, which is involved in the regulation of the immune system [3–5]. Sema4D-deficient mice exhibited functional defects in the immune system [3]. Previous studies found that Sema4D was upregulated in autoimmune diseases and it plays key roles in regulating innate and adaptive immune responses [4, 6, 7]. In addition, Sema4D was found highly and selectively produced by the osteoclasts in bone [8]. It suppressed the differentiation of osteoblast, whereas it did not affect osteoclastogenesis [9]. Circulating Sema4D was increased in rheumatoid arthritis patients, which could serve as a marker for predicting radiographic progression in patients with rheumatoid arthritis [10, 11]. And serum Sema4D is reported to be increased in postmenopausal osteoporosis patients and has an inverse association with lumbar spine bone mineral density, bone alkaline phosphatase, and bone Gla-protein levels [12]. Importantly, Sema4D was markedly increased in the mouse model of temporomandibular arthritis [13]. However, its role in the progress and development of OA is unclear.

Chondrocyte inflammatory responses are known to occur in the development of OA [14, 15]. Lipopolysaccharide (LPS) triggers the immune system, and it is a major factor in the occurrence of osteolytic bone loss [16]. And it is widely adopted to construct cell injury model *in vitro* OA studies [14, 16]. Therefore, an *in vitro* LPS-induced OA-like chondrocyte injury model was constructed in this study. And the effect of Sema4D inhibition and overexpression on the cell viability, apoptosis, and production of proinflammatory cytokines and the underlying mechanisms were explored in the LPS-induced chondrocyte injury model.

The MAPK signaling pathway has been reported to regulate tissue development, homeostasis, and the occurrence and development of diverse human diseases [17]. It participated in many inflammation-related events, for instance, nitric oxide synthase induction, neutrophil activation, apoptosis, and cytokine production [18–21]. This signaling has been considered as potential therapeutic targets for cancer and inflammatory and degenerative diseases such as OA [22, 23]. A recent study found that Sema4D restrained the activation of Erk1/2 in LPS-treated microglia [24]. However, the relationship between Sema4D and the MAPK signaling pathway in LPS-induced chondrocyte injury model is unclear. Hence, the effect of Sema4D on the MAPK pathway was further explored.

## 2. Materials and Methods

### 2.1. Cell Lines

Human cartilage ATDC5 cells were obtained from the Institute of Biochemistry and Cell Biology at the Chinese Academy of Sciences (Shanghai, China). Cells were maintained in DMEM and supplemented with 10% fetal calf serum (Gibco, Rockville, MD).

### 2.2. Cell Viability Test

Cell viability of ATDC5 cells was detected through Cell Counting Kit 8 (CCK-8, Dojindo Laboratories, Kumamoto, Japan) and LDH (lactic dehydrogenase) Assay Kit (Abcam, Cambridge, UK).

#### 2.2.1. CCK-8 Assay Kit

ATDC5 cells were planted into 96-well microplates and cultured overnight. A series of different concentrations of LPS (0, 1, 5, and 10 *μ*g/mL, Sigma-Aldrich, St. Louis, MO, USA) were added into the culture medium and maintained for 12 h. Then, 10 *μ*L CCK-8 reagents were added into each well and cultured in 37°C for 2 h. An Infinite™ M200 microplate reader (Tecan, Mannedorf, Switzerland) was used to measure the optical density (OD) values at 450 nm.

#### 2.2.2. LDH Assay Kit

ATDC5 cells were seeded into 96-well microplates. Following treatment by different concentrations of LPS for 12 h, LDH releasing agent was added and mixed with the culture medium. After incubation for 1 h and centrifugation, the supernatant (100 *μ*L) was transferred to a new microplate. The OD value was measured at 490 nm in the Infinite™ M200 microplate reader.

### 2.3. Apoptosis Assay

Annexin V-FITC Apoptosis Detection Kit (Sigma-Aldrich) was used to analyze the apoptosis rate of ATDC5 cells. Cells were collected and washed by PBS. Then, cells were resuspended in binding buffer solution and mixed with Annexin V reagent. After incubation for 20 min in dark conditions, PI (Propidium Iodide) solution was added and then analyzed by a flow cytometer. The apoptosis rate was calculated by XP032 ADC Analysis software.

### 2.4. RNA Extraction and Quantitative Real-Time PCR

Total RNA from ATDC5 cells were isolated by a TRIzol kit (Invitrogen). Complementary DNA was reverse-transcribed from RNA by M-MLV Reverse Transcriptase. The expression levels of Sema4D, Plexin B1, Plexin B2, and CD72 mRNA were analyzed through quantitative RT-PCR using a SYBR® Premix Ex Taq™ Kit in Thermal Cycler Dice® Real-Time System TP800.

### 2.5. Construct and Transfect of siRNA and Overexpression Vector

siRNAs of Sema4D, Plexin B1, Plexin B2, and CD72 were designed and purchased from RiboBio (Guangzhou, China). Sema4D mRNA sequence was amplified and cloned into pcDNA3.1 vector, empty pcDNA3.1 vector as the negative control (Invitrogen, Carlsbad, CA, USA). Lipofectamine 3000 reagent (Invitrogen) was adopted to transfect siRNAs and overexpression vectors of Sema4D into ATDC5 cells following the manufacturer's instructions.

### 2.6. Western Blotting

ATDC5 cells were scraped and lysed by RIPA lysis buffer (Beyotime Biotechnology, Shanghai, China). After centrifugation at 15000 rpm for 10 min at 4°C, the supernatant was collected and quantified through a BCA Protein Assay Kit (Beyotime Biotechnology). Proteins were electrophoresed in 10% SDS-PAGE and transferred onto PVDF membrane. Following blocking with 5% albumin from bovine serum in PBS, primary antibodies were added and incubated overnight at 4°C. Then, the members were probed with corresponding secondary antibodies at room temperature. The signal of protein bands was visualized through Western blotting detection system (Bio-Rad, USA).

### 2.7. Enzyme-Linked Immunosorbent Assay (ELISA)

The levels of IL-1*β*, IL-6, and TNF-*α* in the culture medium of ATDC5 cells were measured by ELISA kits (Abcam, Cambridge, UK) following the manufacturer's recommendations.

### 2.8. Data Analysis

All data was presented as the mean ± standard deviation (SD). Statistical analysis was performed using one-way analysis of variance (ANOVA) and Student's *t*-test by PRISM 9.0 software (GraphPad, San Diego, California, USA). *p* < 0.05 was considered to indicate a statistically significant result.

## 3. Results

### 3.1. Sema4D Was Increased in LPS-Induced ATDC5 Cells

**Figure 1:**
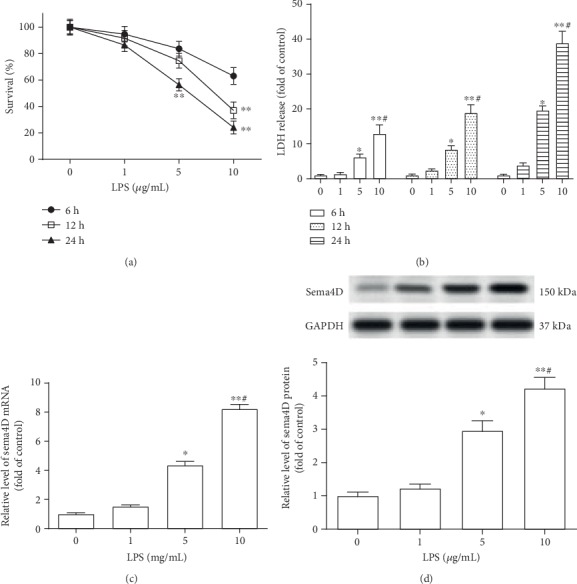
Sema4D was increased in LPS-treated ATDC5 cells. (a) The survival rate of ATDC5 cells was analyzed after treatment with different concentrations of LPS (0, 1, 5, and 10 *μ*g/mL) for 6, 12, and 24 h. (b) The content of LDH in culture medium of ATDC5 cells was examined after stimulation with LPS. (c, d) The expression levels of ATDC5 mRNA and protein were detected through RT-PCR and Western blotting. ATDC5 cells were treated with 5 *μ*g/mL of LPS for 12 h. ^∗^*p* < 0.5 and ^∗∗^*p* < 0.1 versus 0 *μ*g/mL of LPS treatment group; ^#^*p* < 0.5 versus 5 *μ*g/mL of LPS treatment group.

ATDC5 cells were treated by different concentrations of LPS (0, 1, 5, and 10 *μ*g/mL) for 6, 12, and 24 h. The results indicated that 5 and 10 *μ*g/mL LPS significantly suppressed the cell survival rate of ATDC5 cells (Figure 1(a)). Meanwhile, the LDH (lactic dehydrogenase) Assay Kit was adopted to detect the released LDH from damaged ATDC5 cells. As shown in Figure 1(b), the activity of LDH was markedly increased in ATDC5 cells after being treated by LPS (5 and 10 *μ*g/mL). Besides, we found that LPS treatment notably promoted Sema4D expression in ATDC5 cells (Figures 1(c) and 1(d)).

### 3.2. Silence of Sema4D Rescued LPS-Induced Cell Damage in ATDC5 Cells

**Figure 2:**
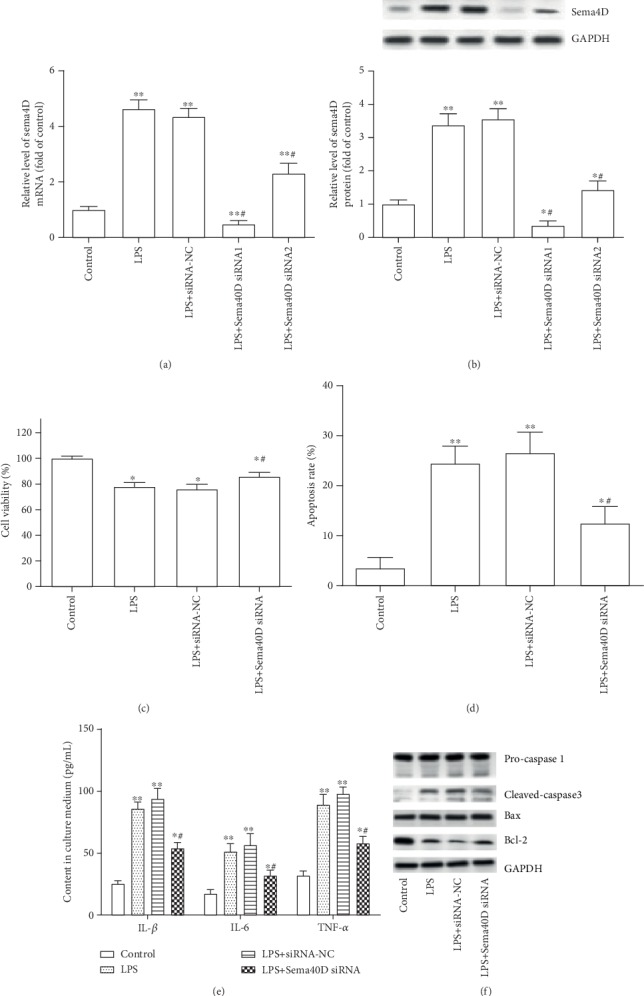
Silence of Sema4D suppressed LPS-induced cell damage in ATDC5 cells. (a, b) The expression of Sema4D mRNA and protein in ATDC5 cells was detected after transfection with siRNAs. ATDC5 cells were transfected with Sema4D siRNA1, Sema4D siRNA2, or siRNA negative control (siRNA-NC); after incubation for 48 h, LPS (5 *μ*g/mL) was added and cultured for 12 h. ^∗^*p* < 0.5 and ^∗∗^*p* < 0.1 versus the control group; ^#^*p* < 0.5 versus the LPS+siRNA-NC treatment group. (c, d) Cell viability and apoptosis of ATDC5 cells were tested. (e) The content of IL-1*β*, IL-6, and TNF-*α* in culture medium of ATDC5 cells was measured through ELISA kits. (f) Expression of pro-caspase 1, cleaved-caspase 3, Bax, and Bcl-2 was detected through Western blotting. ATDC5 cells were transfected with Sema4D siRNA1 and treated with LPS. ^∗^*p* < 0.5 and ^∗∗^*p* < 0.1 versus the control group; ^#^*p* < 0.5 versus the LPS+siRNA-NC treatment group.

To evaluate the effect of Sema4D knockdown on LPS-induced ATDC5 cell injury, ATDC5 cells were pretransfected with Sema4D siRNAs or siRNA-NC for 48 h, then treated by LPS (5 *μ*g/mL) for 12 h. The results show that transfection with Sema4D siRNAs effectively prevented the increase in the expression of Sema4D in LPS-treated ATDC5 cells (Figures 2(a) and 2(b)), while Sema4D siRNA1 exhibited a higher inhibition efficiency in ATDC5 cells than Sema4D siRNA2. Hence, Sema4D siRNA1 was adopted in follow-up experiments. Meanwhile, we found that the inhibition of Sema4D significantly suppressed the declined cell viability in LPS-stimulated ATDC5 cells (Figure 2(c)). Apoptosis assay showed that the knockdown of Sema4D markedly inhibited the cell apoptosis triggered by LPS in ATDC5 cells (Figure 2(d)). In addition, Figure 2(f) shows that pyroptosis biomarker caspase I was not activated after treatment with LPS and si-Sema4D. Apoptosis biomarker caspase-3 was cleaved and activated, and Bcl-2 was downregulated after stimulation by LPS. Inhibition of Sema4D partly suppressed the activation of caspase-3 and the decline of Bcl-2 induced by LPS in ATDC5 cells. Further, the content of proinflammatory factors (IL-1*β*, IL-6, and TNF-*α*) in the culture medium of ATDC5 cells was detected. The results showed that LPS treatment significantly promoted the production of IL-1*β*, IL-6, and TNF-*α* in ATDC5 cells, while the inhibition of Sema4D partly inhibited the increasing secretion of IL-1*β*, IL-6, and TNF-*α* (Figure 2(e)).

### 3.3. Overexpression of Sema4D Exacerbated LPS-Induced Cell Damage

**Figure 3:**
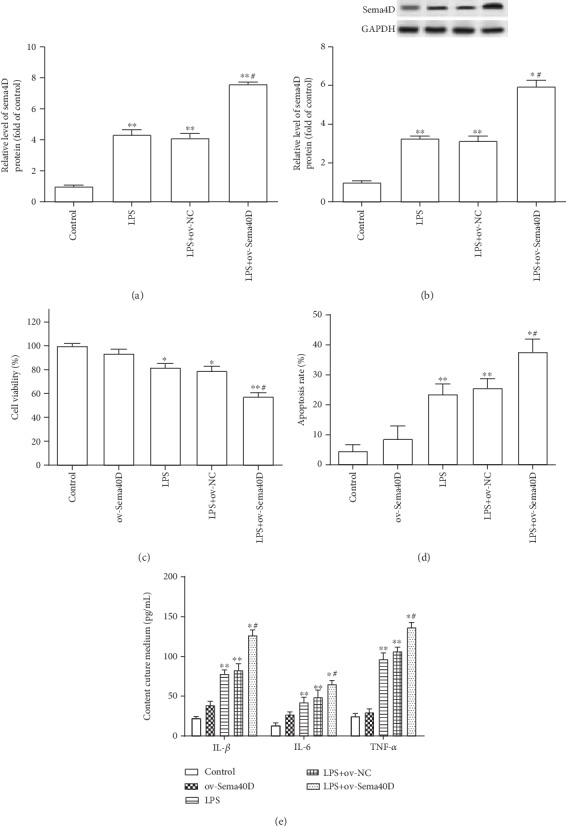
Overexpression of Sema4D exacerbated LPS-induced cell damage in ATDC5 cells. (a, b) The expression of Sema4D mRNA and protein in LPS-treated ATDC5 cells was measured after transfection with overexpression vectors of Sema4D. (c, d) Cell viability and apoptosis of ATDC5 cells were tested. (e) The content of IL-1*β*, IL-6, and TNF-*α* in culture medium of ATDC5 cells was measured through ELISA kits. ATDC5 cells were transfected with overexpression vectors of Sema4D (ov-Sema4D) or empty vectors (ov-NC) and cultured for 48 h. ^∗^*p* < 0.5 and ^∗∗^*p* < 0.1 versus the control group; ^#^*p* < 0.5 versus the LPS+ov-NC treatment group.

Next, overexpression vectors of Sema4D (ov-Sema4D) were transfected into ATDC5 cells. The results showed that ov-Sema4D effectively promoted the expression of Sema4D in LPS-treated ATDC5 cells (Figures 3(a) and 3(b)). Besides, overexpression of Sema4D further promoted the decline of cell viability and apoptosis triggered by LPS treatment in ATDC5 cells (Figures 3(c) and 3(d)). Higher contents of IL-1*β*, IL-6, and TNF-*α* were found in LPS-treated ATDC5 cells after overexpression of Sema4D (Figure 3(e)). Overexpression of Sema4D made no remarkable changes in cell viability, apoptosis, and the secretion of proinflammatory cytokines in ATDC5 cells (Figures 3(c)–3(e)).

### 3.4. Inhibition of the MAPK Signaling Pathway Abolished the Effect of Sema4D Overexpression on LPS-Induced Cell Damage

**Figure 4:**
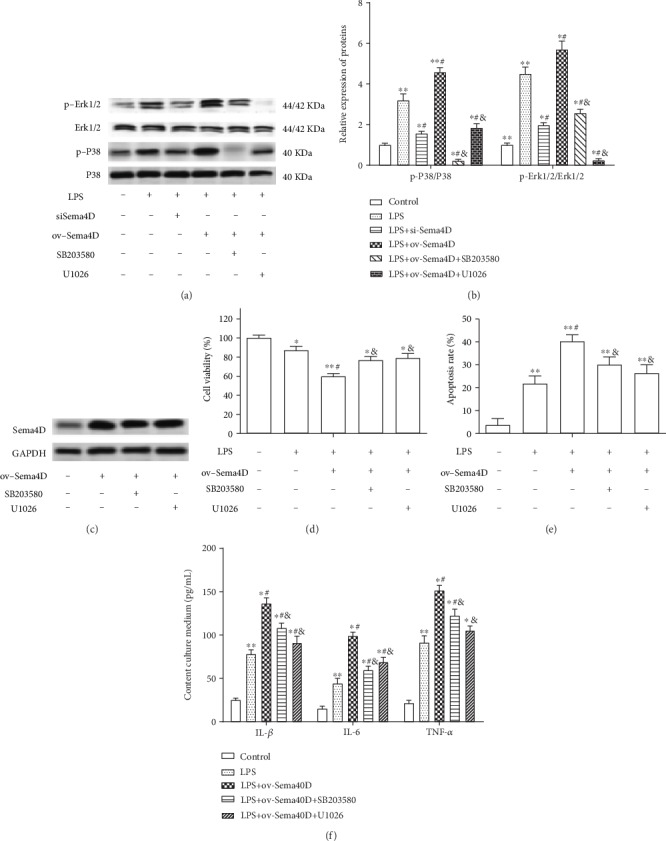
Inhibition of the MAPK signaling pathway abolished the effect of Sema4D on ATDC5 cells. (a, b) The expression of p-P38, P38, Erk1/2, and p-Erk1/2 in ATDC5 cells was examined through Western blotting. (c) The expression of Sema4D in ATDC5 cells was examined after treatment with SB203580 and U1026. (d–f) Cell viability, apoptosis, and levels of IL-1*β*, IL-6, and TNF-*α* were detected. After transfection with ov-Sema4D for 48 h, LPS, SB203580, and U1026 were added and cultured for 12 h. ^∗^*p* < 0.5 and ^∗∗^*p* < 0.1 versus the control group, ^#^*p* < 0.5 versus the LPS treatment group, and ^&^*p* < 0.5 versus the LPS+ov-Sema4D treatment group.

Further, the role of the MAPK signaling pathway in Sema4D-mediated cell apoptosis and inflammation in LPS-stimulated ATDC5 cells was explored. The activation of P38 and Erk1/2 was signally stimulated by LPS in ATDC5 cells (Figures 4(a) and 4(b)). The silence of Sema4D partly suppressed the phosphorylated expression of Erk1/2 and P38 in LPS-stimulated ATDC5 cells (Figures 4(a) and 4(b)), while the overexpression of Sema4D enhanced the expression of p-Erk1/2 and p-P38 in LPS-stimulated ATDC5 cells (Figures 4(a) and 4(b)). The inhibition of the MAPK signaling pathway by SB203580 (P38 inhibitor) or U1026 (Erk1/2 inhibitor) markedly suppressed the increasing expression of p-P38 and p-Erk1/2 in ATDC5 cells induced by LPS and Sema4D overexpression (Figures 4(a) and 4(b)). Nevertheless, SB203580 and U1026 made no significant influence on the level of Sema4D in ATDC5 cells (Figure 4(c)). In addition, we found that the inhibition of the MAPK pathway diminished the acceleration effect of Sema4D overexpression on LPS-induced cell injury (Figures 4(d)–4(f)).

### 3.5. Sema4D Mediated Chondrocyte Damage through Interacting with Its Receptor Plexin B1

**Figure 5:**
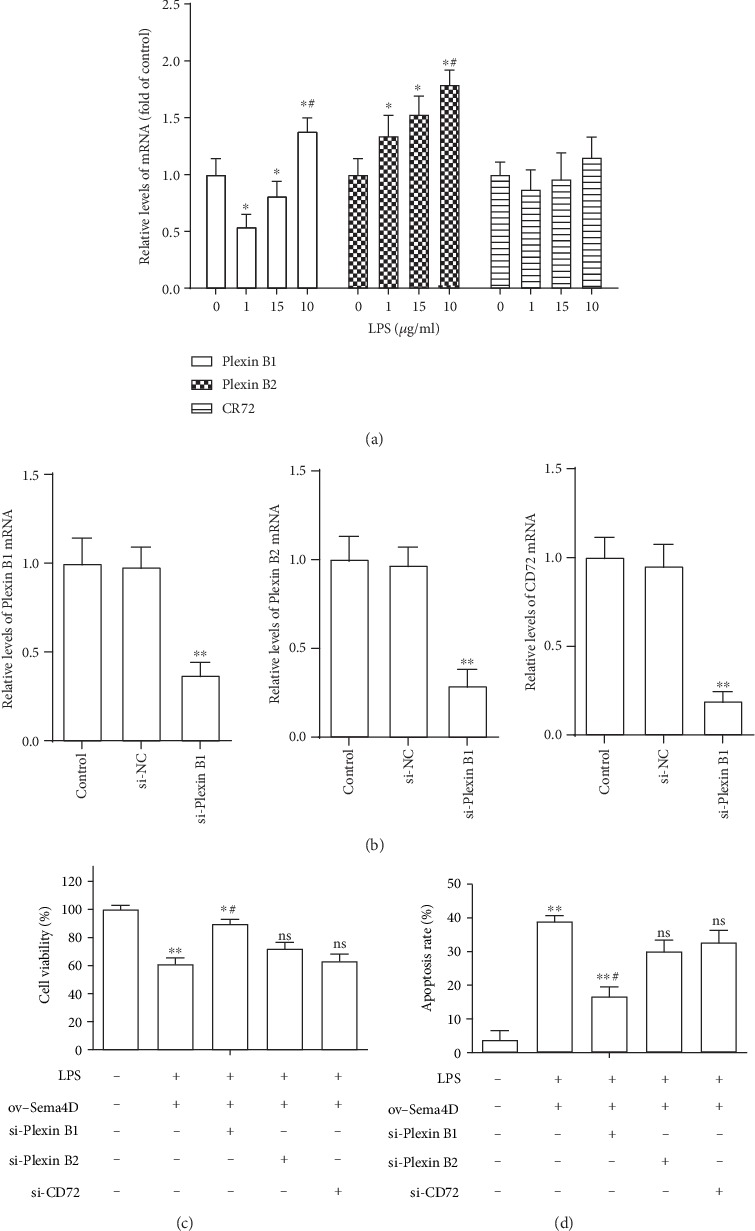
Sema4D mediated cell viability and apoptosis through interacting with its receptor Plexin B1. (a) Relative mRNA levels of Plexin B1, Plexin B2, and CD72 in ATDC5 cells were examined after stimulation with LPS for 12 h. ^∗^*p* < 0.5 and ^∗∗^*p* < 0.1 versus 0 *μ*g/mL of LPS treatment group; ^#^*p* < 0.5 versus 5 *μ*g/mL of LPS treatment group. (b) Expression of Plexin B1, Plexin B2, and CD72 mRNA was tested after transfection with siRNAs. ^∗∗^*p* < 0.1 versus the si-NC transfected group. (c, d) Cell viability and apoptosis were detected after transfection with si-Plexin B1, si-Plexin B2, or si-CD72 in Sema4D-overexpressed LPS-stimulated ATDC5 cells. ^∗^*p* < 0.5 and ^∗∗^*p* < 0.1 versus the untreated group, ^#^*p* < 0.5 versus the LPS+ov-Sema4D treatment group; ns versus no significant difference.

To explore the role of Sema4D receptors Plexin B1, Plexin B2, and CD72 in Sema4D-mediated cell damage in ATDC5 cells, the expression of mRNA levels of Plexin B1, Plexin B2, and CD72 in ATDC5 cells was examined after stimulation by LPS for 12 h. The results showed that the level of Plexin B1 was decreased in the low-dose LPS (1 and 5 *μ*g/mL) treatment group and increased in the 10 *μ*g/mL LPS treatment group (Figure 5(a)). Plexin B1 exhibited upregulation in ATDC5 cells after stimulation by LPS (Figure 5(a)), while CD72 showed no significant changes after treatment by LPS (Figure 5(a)). The inhibition efficiency of si-Plexin B1, si-Plexin B2, and si-CD72 in ATDC5 cells was detected through RT-PCR. The results indicated that si-Plexin B1, si-Plexin B2, and si-CD72 effectively suppressed the expression of Plexin B1, Plexin B2, and CD72 in ATDC5 cells (Figure 5(b)). Next, the effect of deficiency of Plexin B1, Plexin B2, and CD72 on Sema4D-mediated cell damage was detected. As shown in Figures 5(c) and 5(d), the silence of Plexin B1 markedly attenuated Sema4D overexpression-induced decline of cell viability and increase of apoptosis in LPS-stimulated ATDC5 cells, while inhibiting Plexin B2 and CD72 made no remarkable influence on Sema4D overexpression-induced cell damage in LPS-stimulated ATDC5 cells. These results suggest that Sema4D may via interacting with its receptor Plexin B1 regulate LPS-induced chondrocyte damage.

## 4. Discussion

Sema4D is a transmembrane protein, which also exists in a soluble form after the gradual shedding of the Sema4D extracellular domain [25]. In the present study, we found that the expression of membranous Sema4D was increased in ATDC5 cells after stimulation by LPS. Soluble Sema4D level was very low in chondrocyte culture supernatant and has not marked changes after stimulation by LPS. In addition, silence of Sema4D suppressed the declined cell viability and the increased cell apoptosis in ATDC5 cells induced by LPS, while Sema4D overexpression made the opposite effect. These results indicated that Sema4D participated in LPS-induced chondrocyte apoptosis.

Synovitis has been considered as a common feature of OA and correlated with joint dysfunction, cartilage loss, increased severity of symptoms, and increased proinflammatory cytokines [26]. Our study found that the production of IL-1*β*, IL-6, and TNF-*α* was augmented in LPS-treated ATDC5 cells, and this increase was blocked by Sema4D inhibition and promoted by Sema4D overexpression. IL-1*β*, IL-6, and TNF-*α* are proinflammatory cytokines that are reported to play important roles in the progress of OA [27]. Among them, IL-1*β* and TNF-*α* are recognized as the major mediators in the pathophysiology of OA [27]. They are increased in the synovial fluid and membrane of OA patients and triggered the inflammatory cascade [28]. The increased content of IL-6 in serum and synovial fluid was reported to be associated with the severity of lesions in X-ray imaging and knee cartilage loss in OA patients [29], while Sema4D was reported to be upregulated in medication-related osteonecrosis of the jaw and associated with the increased secretion of TNF-*α*, IFN-*γ*, and IL-1*β* [30]. And Sema4D promoted the production of TNF-*α* in macrophages [30]. Sema4D KO mice developed attenuated hypersensitivity responses and lower expression of IL-1*β* and IL-6 [5]. Hence, our study suggested that Sema4D participated in the regulation of the inflammation response in LPS-induced ATDC5 cell injury model.

Increasing evidence demonstrated that the MAPK signaling participated in the occurrence and development of OA [22, 23]. MAPK inhibitors blocked the joint inflammation and destruction in different osteoarthritis animal models [31]. Previous researches have provided ample proofs that the inhibition of the MAPK pathway inhibited the apoptosis and inflammatory response in human OA chondrocytes [22, 32]. On the other hand, Sema4D weakened the phosphorylation of p-Erk1/2 in LPS-treated microglia [24]. On the contrary, another study reported that Sema4D stimulated the increasing expression of p-Erk1/2 in mouse hippocampal HT-22 cells [33]. Our study found that the MAPK signaling pathway was activated in the LPS-induced ATDC5 cell injury model. Sema4D inhibition suppressed the activation of p-P38 and Erk1/2, while Sema4D overexpression promoted the activation of p-P38 and Erk1/2. Importantly, the inhibition of the MAPK pathway abolished the promoting effect of Sema4D overexpression on LPS-induced chondrocyte injury. Hence, our results provide an important insight into the molecular basis of the role of Sema4D in LPS-induced chondrocyte injury. Suppressing Sema4D expression may represent a therapeutic strategy for OA patients.

Accumulating evidence suggested that Sema4D performs its function through its receptors, including Plexin B1, Plexin B2, and CD72 [34]. Plexin B1 was reported as the Sema4D high-affinity receptor in a broad range of nonimmune cells [35]. Sema4D/Plexin B1 interaction participated in angiogenesis, regulating tumor-associated macrophages, and control of invasive growth [36]. Besides, Sema4D/Plexin B1 interactions were reported to be involved in the nervous and immune systems [37]. Plexin B2 is another high-affinity receptor of Sema4D. Sema4D/Plexin B2 interaction could mediate the recruitment and function of T-cell in the germinal center [38, 39] and modulate the adherence between monocytes and endothelial cells of the blood vessels [35]. CD72 is a receptor of Sema4D in lymphoid tissue [40]. Sema4D/CD72 interaction is crucial in activating immune response and maintaining the homeostasis of B-cell antigen receptor signaling [3, 41]. Our study found that the silence of Plexin B1 partly abolished the effect of Sema4D overexpression on LPS-induced chondrocyte injury. What is more, a previous study reported that the knockdown of Plexin B1 suppressed the activation of Erk induced by Sema4D in melanocytes [42]. Hence, it is likely that Sema4D mediates the LPS-induced chondrocyte injury via the MAPK signaling pathway through interacting with Plexin B1.

In summary, our study indicated that Sema4D was increased in LPS-stimulated human cartilage ATDC5 cells. The inhibition of Sema4D suppressed the decline of cell viability and the increasing cell apoptosis and expression of IL-1*β*, IL-6, and TNF-*α* stimulated by LPS. Overexpression of Sema4D aggravated the cell injury triggered by LPS, and inhibiting Plexin B1 partly abolished the effect of Sema4D overexpression on LPS-induced chondrocyte injury. What is more, the MAPK signaling pathway was activated in LPS-stimulated ATDC5 cells. The inhibition of the MAPK signaling pathway abolished the effect of Sema4D on ATDC5 cells. Therefore, this study suggested that Sema4D mediates the LPS-induced chondrocyte injury via regulating the MAPK signaling pathway through interacting with Plexin B1.

## Data Availability

The data used to support the findings of this study are included within the article.
